# Single Nucleotide Polymorphism Effects on Lamb Fecal Egg Count Estimated Breeding Values in Progeny-Tested Katahdin Sires

**DOI:** 10.3389/fgene.2022.866176

**Published:** 2022-05-03

**Authors:** David R. Notter, Marzieh Heidaritabar, Joan M. Burke, Masoud Shirali, Brenda M. Murdoch, James L. M. Morgan, Gota Morota, Tad S. Sonstegard, Gabrielle M. Becker, Gordon L. Spangler, Michael D. MacNeil, James E. Miller

**Affiliations:** ^1^ Department of Animal and Poultry Sciences, Virginia Tech, Blacksburg, VA, United States; ^2^ Livestock Gentec, Department of Agricultural, Food, and Nutritional Science, University of Alberta, Edmonton, AB, Canada; ^3^ United States Department of Agriculture, Agricultural Research Service, Dale Bumpers Small Farms Research Center, Booneville, AR, United States; ^4^ Agri-Food and Biosciences Institute, Belfast, United Kingdom; ^5^ School of Biological Sciences, Queen’s University Belfast, Belfast, United Kingdom; ^6^ Department of Animal, Veterinary and Food Sciences, University of Idaho, Moscow, ID, United States; ^7^ Katahdin Hair Sheep International, Fayetteville, AR, United States; ^8^ Acceligen, Eagan, MN, United States; ^9^ Animal Genomics and Improvement Laboratory, United States Department of Agriculture, Agricultural Research Service, Beltsville, MD, United States; ^10^ Delta G, Miles City, MT, United States; ^11^ Department of Animal, Wildlife and Grassland Sciences, University of the Free State, Bloemfontein, South Africa; ^12^ Department of Pathobiological Sciences, School of Veterinary Medicine, Louisiana State University, Baton Rouge, LA, United States

**Keywords:** fecal egg counts, gastrointestinal parasites, genome-wide association, parasite resistance, regional heritability mapping, sheep

## Abstract

Estimated breeding values (EBV) for fecal egg counts (FEC) at 42–90 days of age (WFEC) and 91–150 days of age (PFEC) for 84 progeny-tested Katahdin sires were used to identify associations of deregressed EBV with single-nucleotide polymorphisms (SNP) using 388,000 SNP with minor-allele frequencies ≥0.10 on an Illumina high-density ovine array. Associations between markers and FEC EBV were initially quantified by single-SNP linear regression. Effects of linkage disequilibrium (LD) were minimized by assigning SNP to 2,535 consecutive 1-Mb bins and focusing on the effect of the most significant SNP in each bin. Bonferroni correction was used to define bin-based (BB) genome- and chromosome-wide significance. Six bins on chromosome 5 achieved BB genome-wide significance for PFEC EBV, and three of those SNP achieved chromosome-wide significance after Bonferroni correction based on the 14,530 total SNP on chromosome 5. These bins were nested within 12 consecutive bins between 59 and 71 Mb on chromosome 5 that reached BB chromosome-wide significance. The largest SNP effects were at 63, 67, and 70 Mb, with LD among these SNP of *r* ^2^ ≤ 0.2. Regional heritability mapping (RHM) was then used to evaluate the ability of different genomic regions to account for additive variance in FEC EBV. Chromosome-level RHM indicated that one 500-SNP window between 65.9 and 69.9 Mb accounted for significant variation in PFEC EBV. Five additional 500-SNP windows between 59.3 and 71.6 Mb reached suggestive (*p* < 0.10) significance for PFEC EBV. Although previous studies rarely identified markers for parasite resistance on chromosome 5, the *IL12B* gene at 68.5 Mb codes for the p40 subunit of both interleukins 12 and 23. Other immunoregulatory genes are also located in this region of chromosome 5, providing opportunity for additive or associative effects.

## 1 Introduction

Improvement in genetically mediated resistance to gastrointestinal nematodes (GIN) is a potentially important component of an integrated management strategy to reduce the impact of parasitism in small ruminants (Maqbool et al., 2017). Genetic evaluation of GIN resistance is relatively straightforward in areas of high parasite challenge, most commonly using measurements of fecal egg counts (FEC) in young lambs to derive estimated breeding values (EBV) that can be used in the selection of breeding animals (Brown et al., 2007; Notter, 2013).

The Katahdin breed was derived in the US from crosses among Caribbean hair sheep and various temperate wool breeds (Wildeus, 1997). The Katahdin has greater GIN parasite resistance than other US breeds (Vanimisetti et al., 2004) and significant levels of additive genetic variation in FEC in lambs at weaning and postweaning ages (Ngere et al., 2018). As a result, resistance to GIN parasites has become a signature trait of this breed, especially in the warm, humid conditions that are typical of the southeastern United States. GIN parasites are endemic in most flocks in this region, allowing direct measurements of FEC to be used for genetic evaluation of parasite resistance. However, the Katahdin breed has several “easy-care” traits in addition to parasite resistance (e.g., freedom from wool, high twinning rates, good mothering ability) and has led the US sheep industry in numbers of sheep registered and transferred for a majority of the last 10 years. As a result, Katahdins have expanded in recent years into areas of less consistent parasite challenge, and Katahdin flocks are now found in areas where genetic evaluation of GIN resistance using phenotypic measurements of FEC is not always possible. This situation is not a concern for flocks that have customers located only in arid regions that rarely experience high levels of parasitism. However, many potential customers are attracted to the Katahdin breed for their resilience to sporadic and less predictable parasite challenges. Some Katahdin breeders also wish to ensure that they can market animals in areas of both high and low parasite challenge.

In common with other disease and fitness traits, genome-based selection appears to be a valuable option for screening prospective breeding animals for parasite resistance in regions or years when parasite challenge is not adequate to support genetic evaluations based only on phenotypic measurements (Bishop and Woolliams, 2014). Use of genomic information also allows breeders who cannot, or prefer to not, collect FEC to evaluate their animals for parasite resistance.

A substantial number of studies have attempted to identify genetic markers for GIN parasite resistance (see Benavides et al., 2016 for review and references in Table 1). These studies have involved both tropical hair sheep (e.g., Marshall et al., 2012; Berton et al., 2017) and temperate wool breeds (e.g., Riggio et al., 2013; Pickering et al., 2015; Al Kalaldeh et al., 2019) and consistently identified significant or suggestive associations between various sorts of genetic markers and measures of parasite resistance such as FEC, packed cell volume, Faffa Malan chart (FAMACHA) scores, and circulating immunoglobulin levels in response to artificial or natural GIN infection. However, marker studies involving either specific candidate genes and genomic regions or, more recently, large-scale screening of the entire genome using single-nucleotide polymorphisms (SNP) have not identified consistent associations with parasite resistance, even in studies involving the same breeds (e.g., Silva et al., 2011; Marshall et al., 2012; Becker et al., 2020; Becker et al., 2022). These inconsistencies suggest that large, breed-specific studies will be necessary to identify sets of genetic markers that can be relied upon to predict FEC EBV with acceptable reliability. This situation is further exacerbated by the large number of global sheep breeds, their often-modest population sizes, and the diverse production conditions that are typical of small ruminant production.

**TABLE 1:** Candidate genes.

Candidate gene	Location^a^	References^b^
Interferon gamma (*IFNG*)	3: 151.8	Beh et al. (2002)
		Coltman et al. (2001)
		Dominik et al. (2010)
		Marshall et al. (2009)
		Sallé et al. (2012)
MHC DR beta chain (*DRB1*)	20: 25.4	Davies et al. (2006)
		Janßen et al. (2002)
		Marshall et al. (2009)
		Sallé et al. (2012)
		Schwaiger et al. (1995)
Toll-like receptors (*TLR*) 1, 6, and 10	6: 58.7	Beh et al. (2002)
		Gutiérrez-Gil et al. (2009)
DIS3-like 3′-5′ exoribonuclease 2 (*DIS3L2*)	2: 233.6	Becker et al. (2020)
Interferon regulatory factor 2 binding protein 2 (*IRF2BP2*)	25: 6.6	Demars et al. (2017)

a Chromosome: Mb.

Progeny-tested sires with high-accuracy EBV provide a unique resource for study of the association between genomic markers and animal breeding values. These sires are normally the most accurately evaluated individuals in the breed and are anticipated to have the greatest impact on future generations. The current study therefore utilized a group of progeny-tested Katahdin sires to assess associations between SNP markers and FEC EBV. The study used a two-step strategy for this analysis. Step 1 (the validation phase) involved the evaluation of five *a priori* candidate regions of the genome that were previously implicated in GIN parasite resistance. Step 2 (the discovery phase) then applied a genome-wide association study (GWAS) to identify previously unknown or less commonly reported regions that appeared to influence parasite resistance in these Katahdin sires.

## 2 Materials and Methods

### 2.1 Sampling and Selection of Sires

Estimated breeding values (EBV) for fecal egg counts (FEC) at 42–90 days of age (weaning FEC EBV; WFEC) and 91–150 days of age (postweaning FEC EBV; PFEC) for progeny-tested Katahdin sires from the US National Sheep Improvement Program (NSIP) were screened to identify sires with prediction accuracies (i.e., predicted correlations between actual and predicted breeding values) of 0.60 or more for WFEC or PFEC.

Fecal samples were collected from the rectums of grazing Katahdin lambs, refrigerated for up to 7 days, and sent to the Virginia-Maryland Regional College of Veterinary Medicine or the Louisiana State University School of Veterinary Medicine for determination of FEC using the McMaster method (Whitlock, 1948). Samples taken at 42–90 days of age came from suckling lambs. Samples taken at 91–150 days of age came from a mixture of weaned and unweaned lambs, depending on the management of the participating flocks. Management groups were defined by the flock, year, season (based on 30-d rolling birth date windows), and lamb sex, and an optional producer-supplied management code that was used to identify other flock-specific management differences among otherwise contemporary lambs. The FEC were recorded as eggs per gram of feces (epg) but normalized by cube-root transformation before analysis, and FEC EBV on the cube-root scale were derived from a multi-trait animal model (Brown et al., 2007). Estimated heritabilities were 0.27 for WFEC and 0.50 for PFEC, and corresponding additive genetic and residual correlations between WFEC and PFEC were 0.81 and 0.07, respectively (Notter, 2013). No other additive direct or maternal correlations between WFEC or PFEC and other traits were included in the prediction model. Resulting FEC EBV on the cube-root scale were further transformed for reporting to a predicted percentage of the mean FEC EBV, but analyses for the current study used FEC EBV expressed on the cube-root scale. See Notter (2013) and Brown et al. (2007) for additional details of NSIP procedures for recording and analysis of FEC and reporting of FEC EBV.

Screening of Katahdin EBV released on 15 September 2015, identified 329 progeny-tested sires with accuracies of FEC EBV of 0.60 or greater for WFEC and (or) PFEC, and 86 of these rams were located for DNA sampling. Eight additional young rams that were anticipated to produce progeny in 2016 were also sampled. DNA samples were obtained from archived DNA (Gene Check, Inc., Greeley, CO) or from ear tissue or jugular blood that was processed using standard laboratory procedures at the USDA ARS Beltsville Agricultural Research Center, Beltsville, MD. Ten rams were subsequently excluded from the study based on the yield and quality of the DNA, the presence of inconsistencies in the pedigree file, or because anticipated data from 2016 lambs were not submitted to NSIP, leaving 84 progeny-tested rams for further analysis. The FEC EBV for these sires were based on FEC records for 3,272 lambs from 14 flocks. Of these lambs, 1,469 had both weaning and postweaning FEC records, 647 had only WFEC, and 1,156 had only PFEC. The median number of progeny with FEC data was 31 per sire, with a range of 3–149 lambs. Most sires had progeny in only one flock, but 11 sires each had progeny in two flocks, two sires each had progeny in three flocks, and three sires each had progeny in four flocks. Comparisons of EBV for rams evaluated in different flocks therefore in most cases relied on pedigree connections among the flocks.

Based on NSIP Katahdin EBV released on 17 January 2019, means for WFEC and PFEC EBV for the sampled sires were -8.3 and -14.3%, respectively, with ranges of -96.6–241.5% for WFEC and -99.9–387.9% for PFEC. Averages (ranges) for accuracies of FEC EBV were 0.82 (0.50–0.94) for WFEC and 0.86 (0.52–0.96) for PFEC. If reported FEC EBV expressed on the cube-root scale were converted to the original (epg) scale, the reported FEC EBV ranges corresponded to ranges in EBV on the original scale of −483–1,208 epg for WFEC and −499–1,939 epg for PFEC.

### 2.2 Laboratory Methods

The High-Density (600 K) Illumina Ovine SNP BeadChip (Illumina, Inc., San Diego, CA, United States; Kijas et al., 2014) was applied to DNA from the 84 progeny-tested sires. Raw data were analyzed using GenomeStudio V2011.1 and Genotyping Module V.1.9.4. All the 84 rams had call rates of >99% and were included in the final analyses.

### 2.3 Statistical Methods

**FIGURE 1:**
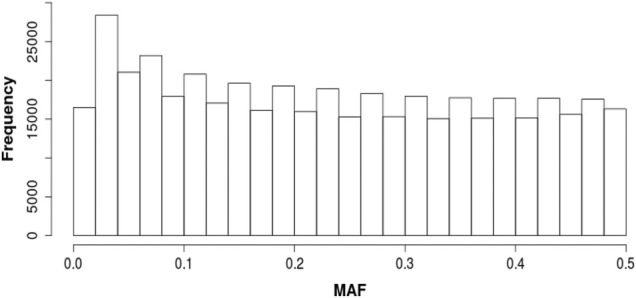
Distribution of minor allele frequencies (MAF).

The SNP used for this study were required to have minor allele frequencies (MAF) of at least 0.10. This minimum MAF was chosen, in part, because of the small number of sires in the study. A SNP allele with MAF of 0.10 has an expected frequency of homozygous individuals of 0.01, corresponding to less than one individual in our sample of 84 sires. Also, because the Katahdin is a relatively recently formed composite breed, we wished to focus on SNP alleles and associated quantitative trait locus (QTL) alleles that were likely to have originated in the different parental breeds and anticipated to still be segregating at high frequencies. The distribution of MAF before removing alleles with MAF <0.10 is shown in Figure 1. After screening on MAF, an additional 163 duplicate variants were identified and removed, leaving approximately 388,000 SNP for use in the analysis.

The effect of each SNP was initially evaluated by regressing WFEC and PFEC EBV on the number of occurrences of the most common allele for each SNP (0, 1, or 2) using a single-marker GWAS model. Before analysis, FEC EBV on the cube-root scale were deregressed, to adjust for differences in accuracy of prediction of EBV using procedure described by Garrick et al. (2013). An additive pedigree relationship matrix (A) was incorporated into the analysis using a pedigree file (*n* = 737) derived from NSIP Katahdin data. The statistical model for each SNP and FEC EBV was:

Y = Xβ + Za + e (1)

where Y is a vector of deregressed FEC EBV, β is a vector containing estimates of the intercept and marker effect, a is a vector of additive polygenic random effects with a ∼ N(0, A $\sigma_{a}^{2}\;$ ), X and Z are incidence matrices for β and a, respectively, and e is a vector of residuals. The model was fitted using ASReml software (v. 4.1; Gilmour et al., 2014).

The small number of progeny-tested rams and large number of SNP led to high risk of type I error. Interpretation of results therefore initially focused on five regions of the genome that were considered to have high *a priori* probabilities of containing previously reported genes associated with resistance to gastrointestinal nematodes (Table 1). Three of these regions, identified from the literature, were associated with the interferon gamma gene (*IFNγ*) on ovine chromosome 3 (Coltman et al., 2001; Beh et al., 2002; Marshall et al.,2009; Sallé et al., 2012; Dominik et al., 2010); the gene for the MHC DR beta chain (*DRB1*) within the ovine major histocompatibility complex on chromosome 20 (Schwaiger et al., 1995; Janßen et al., 2002; Davies et al., 2006; Marshall et al., 2009; Sallé et al., 2012); and a region on chromosome 6 that included genes for toll-like receptors (*TLR*) 1, 6, and 10 (Beh et al., 2002; Gutiérrez-Gil et al., 2009). A fourth candidate region on chromosome 2 was associated with FEC in a sample of Katahdin lambs and contained the DIS3 like 3′-5′ exoribonuclease 2 gene (*DIS3L2*; Becker et al., 2020). The final candidate region was located at 6.8 Mb on chromosome 25 and suggested as a candidate region for parasite resistance by G. L. Spangler (unpublished) based on a mutation in the interferon regulatory factor 2 binding protein 2 (*IRF2BP2*) gene that was associated with a hair coat in a hair x wool composite sheep breed (Demars et al., 2017). A nearby gene for an RNA regulatory element, TAR (HIV-1) RNA Binding Protein 1 (*TARBP1*) has been associated with human atutoimmune diseases (Towns and Begley, 2012) and is located at 6.6 Mb on ovine chromosome 25. Each candidate region was defined by a 1-Mb region surrounding the most likely candidate gene(s) in each region (Table 1). To analyze effects of these candidate regions, the genome was subdivided into 2,540 adjacent “bins” of approximately 1 Mb. The effect of a candidate region on FEC EBV was defined as the effect of the most significant individual SNP in the region and tested using Model 1 at *p* < 0.05 following Bonferroni correction of *p* = 0.05/5 = 0.01.

After testing effects of the *a priori* regions, bins containing these regions were excluded from further consideration. Screening of SNP effects on WFEC and PFEC for the remaining 2,535 bins was based on the most-significant SNP within each bin. A Bonferroni correction of *p* = 0.05/2535 = 1.96 × 10^–5^ was used to define bin-based (BB) genome-wide significance. Bin-based chromosome-wide significance was defined as *p* = 0.05/b_i_ where b_i_ was the number of non-candidate bins on chromosome i.

The final step in the analysis involved regional heritability mapping (RHM; Nagamine et al., 2012; Shirali et al., 2016) to assess the impact of individual regions of the genome on additive variance in deregressed FEC using Genome-wide Complex Trait Analysis (GCTA) software (version 1.93.0; Yang et al., 2011). For RHM, a series of windows containing 1 Mb, 100 sequential SNP, or 500 sequential SNP were selected, with 0.5 Mb, 50 SNP, or 250 SNP overlaps, respectively, between adjacent windows. A whole-genome relationship matrix was constructed from SNP with MAF ≥0.1 using GCTA, and regional genomic relationship matrixes were constructed for each window. Contributions of SNP effects in each window to FEC EBV were evaluated as:

Y = μ + Zg + Qr + e (2)

where Y is a vector of deregressed FEC EBV, μ is the population mean, Z and Q are incidence matrices for the whole (without the window) and the regional random effects, respectively, and **e** is a vector of residuals. The distributions and covariance structures of g and r were g ∼ N(0, G $\sigma_{g}^{2}$ ) and r ∼ N(0, $G_{r}\sigma_{r}^{2}$ ), respectively, where G is the whole genomic relationship matrix, $G_{r}$ is the regional genomic relationship matrix, $\sigma_{g}^{2}$ is the whole additive genetic variance, and $\sigma_{r}^{2}$ is the regional additive genetic variance. The impact of each region was tested in GCTA using a likelihood-ratio test to compare Model 2 with a null model which excluded regional SNP effects. Genome-wide (*p* ≤ 0.05) and suggestive (*p* ≤ 0.10) significant levels for each region were determined after Bonferroni correction based on the number of non-overlapped windows.

A single-marker GWAS was also conducted using GCTA with a genomic relationship matrix constructed using SNP with MAF ≥0.1. Each SNP was fitted as a fixed effect using Model 1, and results were compared with those obtained from the bin-based analysis. Genome-wide significance was tested for each SNP at *p* ≤ 0.05 after Bonferroni correction based on the 388,000 SNP with MAF ≥0.10.

## 3 Results

### 3.1 Evaluation of Candidate Regions

None of the SNP within the five candidate regions had even a nominally significant association with FEC EBV (*p* > 0.10).

### 3.2 Bin-Based Genome-Wide Association Study for Non-Candidate Regions

#### 3.2.1 Tests of Significance

**FIGURE 2:**
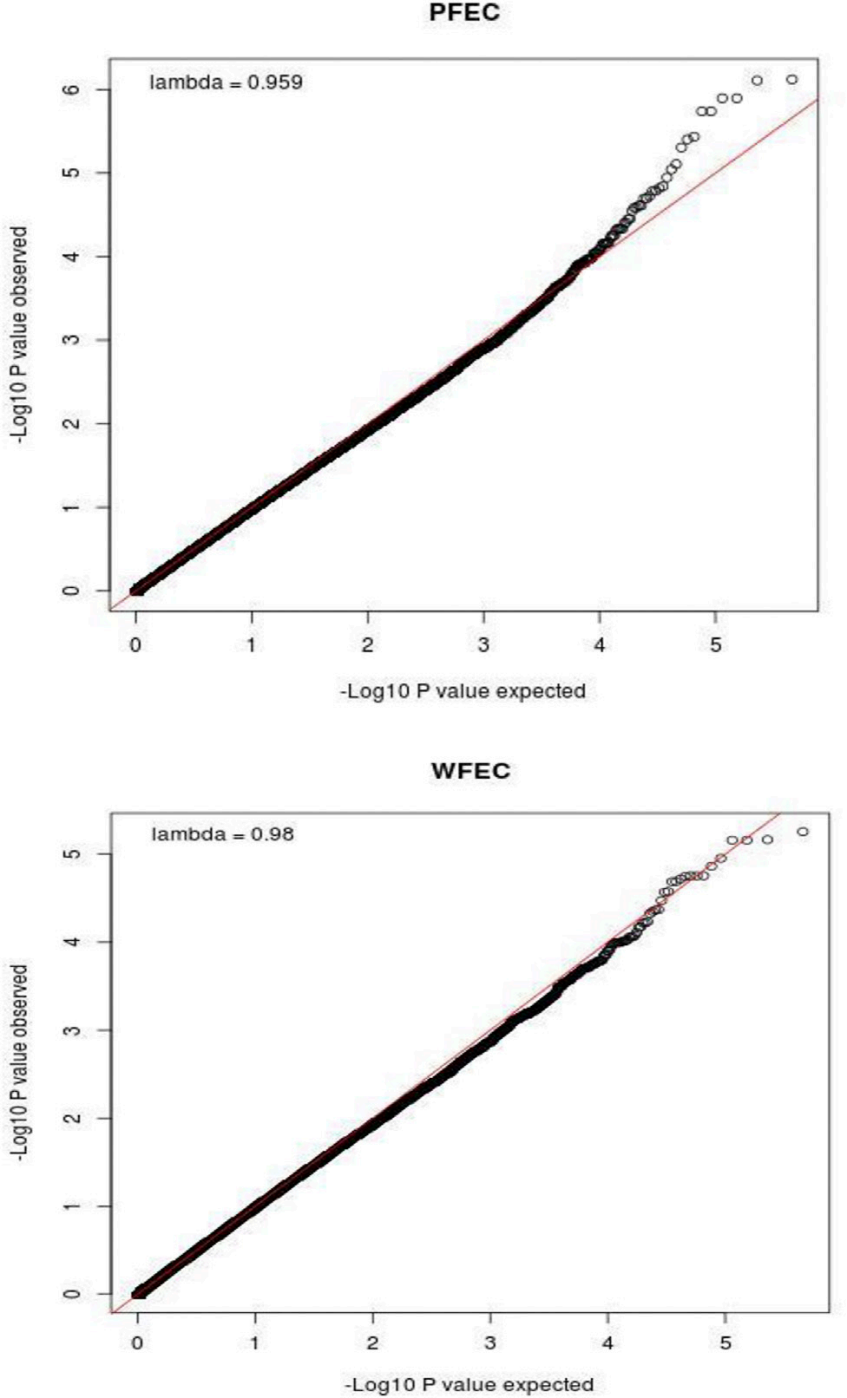
QQ plots showing the relationship between observed and expected values for -Log_10_(P) for postweaning (PFEC) and weaning (WFEC) fecal egg count EBV from the single-SNP genome-wide association study using a pedigree-based relationship matrix. P is the nominal significance level for each SNP.

For WFEC, four bins on chromosome 5 and two bins on chromosome 16 achieved BB genome-wide significance, and an additional 59 bins contained SNP with BB chromosome-wide significance. These bins included four additional bins on chromosome 5 and four additional bins on chromosome 16. The remaining 51 bins with BB chromosome-wide significance were scattered among 20 chromosomes, and none were adjacent. For PFEC, six bins on chromosome 5 achieved BB genome-wide significance. No other bins reached this level of significance. However, 48 additional bins, including 13 bins on chromosome 5 and seven bins on chromosome 16, had at least one SNP with BB chromosome-wide significance for PFEC. The remaining 28 bins that reached BB chromosome-wide significance for PFEC EBV were scattered among 16 different chromosomes, and none were adjacent. QQ plots of the distribution of significance levels for SNP for PFEC and WFEC are shown in Figure 2. Based on these patterns of BB significance, further analysis was restricted to sites on chromosomes 5 and 16.

Several options for SNP pruning or clumping (Marees et al., 2018) were considered in PLINK as alternatives to the bin-based approach. However, as reported by Kijas et al. (2014) for other sheep breeds, generally low levels of LD were observed among SNP in the HD Illumina array. As a result, the pruning and clumping strategies produced only modest reductions in the effective number of independent SNP, yielded Bonferroni-corrected significance levels intermediate to those obtained for the bin-based and individual-SNP analyses and will not be discussed further.

#### 3.2.2 Associations Between FEC EBV and Single-Nucleotide Polymorphisms on Chromosome 5

**FIGURE 3:**
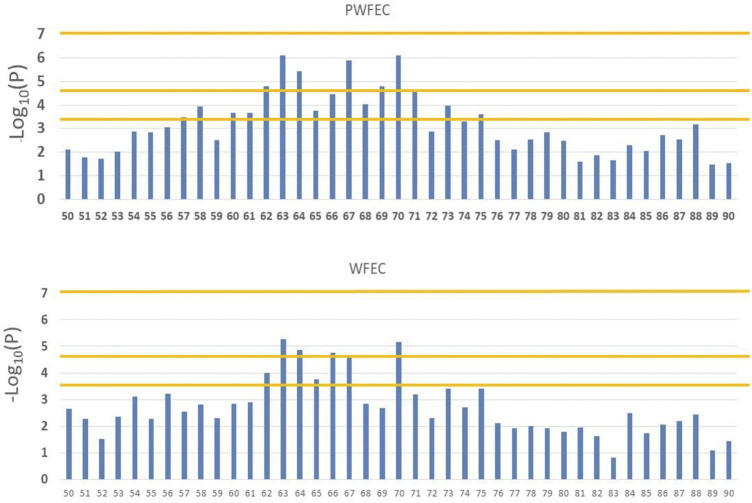
Manhattan plots for associations between SNP in bins 50 through 90 on chromosome 5 and weaning (WFEC) and postweaning (PFEC) fecal egg count EBV. Horizontal lines indicate chromosome-wide significance with 2,540 bins (bottom line), genome-wide significance with 2,540 bins (middle line), and genome-wide significance with 458,625 SNP (top line).

For PFEC EBV (Figure 3), the 19 bins on chromosome 5 that achieved BB chromosome-wide significance included 12 consecutive bins between 59.6 and 71.0 Mb. These bins were imbedded in a larger region that contained 16 bins with BB chromosome-wide significance in a total of 19 bins between 56.1 and 75.0 Mb. Nominal significance levels for the 16 bins ranged from 7.56 × 10^–7^ to 3.46 × 10^–4^. For WFEC EBV (Figure 3), eight bins on chromosome 5 contained at least one SNP with BB chromosome-wide significance and included six consecutive bins that were a subset of the 12 bins that contained a SNP with BB chromosome-wide significance for PFEC EBV. The eight total bins on chromosome 5 that reached BB chromosome-wide significance for WFEC EBV were likewise nested within the 19-bin region identified for PFEC EBV.

Three SNP on chromosome 5 reached chromosome-wide significance at *p* < 0.05 for PFEC EBV after a more conservative Bonferroni correction based on the 14,530 SNP with MAF ≥0.10 on chromosome 5 (i.e., *p* = 0.05/14,539 = 3.44 × 10^–6^), but none of the SNP on chromosome 5 reached this level of significance for WFEC EBV. Genome-wide significance after Bonferroni correction based on the total of 388,000 SNP with MAF ≥0.10 (*p* < 1.29 × 10^–7^) was not achieved for any individual SNP; the most significant SNP associated with PFEC had a nominal significance level of *p* = 7.56 × 10^–7^.

Individual tests of significance did not consider clumping of SNP with large effects on chromosome 5. After excluding SNP from chromosomes 5 and 16 (i.e., those considered to potentially contain SNP with significant associations with FEC EBV), the probability of obtaining BB chromosome-wide significance for PFEC EBV for two adjacent bins was 1.27 × 10^–3^ for the remaining 2,355 bins. Three adjacent bins with BB chromosome-wide significance did not occur. The estimated likelihood of obtaining 12 consecutive bins with BB chromosome-wide significance was therefore (1.27 × 10^–3^)^11^ = 1.39 × 10^–32^. This result suggested that the pattern of SNP effects on chromosome 5 differed from that expected under the assumption of a random pattern of BB chromosome-wide significance among adjacent bins. For WFEC EBV, after excluding bins on chromosomes 5 and 16, the probability of BB chromosome-wide significance for pairs of adjacent bins was 4.66 × 10^–3^, and the predicted probability of obtaining six consecutive bins with BB chromosome-wide significance was (4.66 × 10^–3^)^5^ = 2.20 × 10^–12^. These results indicated an association with FEC EBV across six to 12 adjacent bins on chromosome 5.

Minor allele frequencies for SNP with the highest nominal significance levels in the 12 bins on chromosome 5 shown in Figure 3 averaged 0.30 and ranged from 0.11 to 0.44, with MAF for 11 of the 12 SNP of >0.21. The average frequency of homozygotes for the minor alleles was 0.33 and ranged from 0.04 to 0.70. The SNP effects for these loci were all negative, indicating that the minor allele was consistently favorable (i.e., reduced FEC EBV).

**FIGURE 4:**
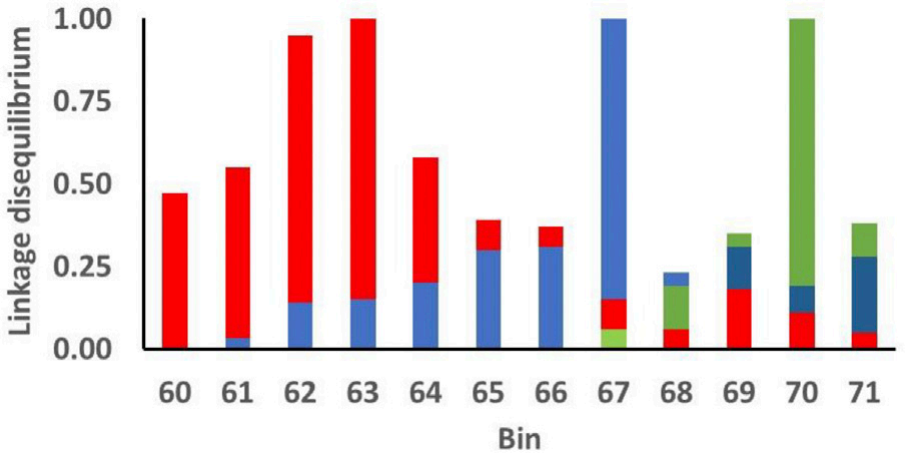
Linkage disequilibrium (*r* ^2^) among the most significant markers for postweaning fecal egg count estimated breeding values (PFEC EBV) in each of the indicated 1-Mb bins on chromosome 5. The *x*-axis indicates the position (Mb) of each bin on chromosome 5. Bins originating at 63, 67, and 70 Mb contained the individual SNP markers with the most significant associations with the PFEC EBV (Figure 3). For each color, the top of the bar of that color for each bin designates the linkage disequilibrium between the most significant marker in the bin and the most significant marker in bins 63 (red), 67 (blue), and 70 (green). For example, the most significant SNP in bin 71 has LD of 0.04 with the most significant SNP in bin 63 (top of the red bar), LD of 0.28 with the most significant SNP in bin 67 (top of the blue bar), and 0.35 with the most significant SNP in bin 70 (top of the green bar).

Linkage disequilibrium (LD) among SNP and associated functional loci can account for clustering of significant SNP. Use of BB statistics reduced, but did not necessarily remove, potential effects of LD among SNP in adjacent bins. For the 12 consecutive bins on chromosome 5 that exhibited BB chromosome-wide significance for PFEC EBV (Figure 3), the largest effects were exhibited for SNP in bins located at 63, 67, and 70 Mb, with lower significance levels for SNP in surrounding bins. Figure 4 shows LD between the most-significant SNP in each of these 12 bins and the most-significant SNP in bins 63, 67, and 70. In agreement with previous results from this high-density ovine SNP array (Kijas et al., 2014), LD on chromosome 5 declined relatively rapidly as the distance between markers increased. The LD between the most-significant markers in bins 63, 67, and 70 were *r* ^2^ = 0.2 or less, indicating that associations between SNP in these regions and PFEC EBV were not explained by LD among the 12 most relevant markers and associated LD of these markers with a single large functional variant. Instead, the observed patterns of LD and associated significance levels for these markers suggested the presence of three functional sites within the 12 bins. Low levels of LD for these markers also indicated that an attempt to identify unique marker haplotypes across this 12-bin region would not be informative.

#### 3.2.3 Associations Between FEC EBV and Single-Nucleotide Polymorphisms on Chromosome 16

Justification for significant effects on FEC EBV for SNP on chromosome 16 was less compelling than that for SNP on chromosome 5. However, BB chromosome-wide significance across adjacent SNP on chromosome 16 and the tendency for SNP on chromosome 16 with BB chromosome-wide significance to have a greater influence on WFEC, compared to PFEC EBV, motivated further consideration of SNP effects on chromosome 16. Bin-based chromosome-wide significance was observed for WFEC EBV for four adjacent bins located between 46.9 and 50.0 Mb on chromosome 16, with nominal significance levels of 1.91 × 10^–5^–2.01 × 10^–4^. Two of these bins reached BB genome-wide significance. Three adjacent bins in the interval reached BB chromosome-wide significance for PFEC EBV, with nominal significance levels of 2.34 × 10^–4^–4.38 × 10^–4^. None of these SNP individually reached genome-wide significance based on the 388,000 SNP with MAF ≥0.10 or chromosome-wide significance based on the 9,749 SNP with MAF ≥0.10 on chromosome 16. However, based on probabilities of obtaining adjacent SNP with BB chromosome-wide significance defined in the preceding section, the likelihood of obtaining four consecutive bins with BB chromosome-wide significance for WFEC EBV was (4.66 × 10^–3^)^3^ = 1.01 × 10^–8^. The likelihood of obtaining three consecutive bins with BB chromosome-wide significance for PFEC EBV was (1.27 × 10^–3^)^2^ = 1.61 × 10^–6^. These results suggest a possible association with WFEC EBV across four bins on chromosome 16 but, at best, only a marginal association with PFEC EBV for three adjacent bins in the same region.

### 3.3 Regional Heritability Mapping

Estimates of SNP-based genomic heritabilities for deregressed FEC EBV from Model 2 were 0.90 ± 0.23 for WFEC (*p* = 0.001) and 0.87 ± 0.23 (*p* = 0.001) for PFEC. The high estimates of heritability were consistent with the high accuracies of the FEC EBV. For an average EBV accuracy of 0.84, the corresponding heritability of the EBV (i.e., of the progeny mean FEC) was expected to be the square root of the accuracy, or 0.92, which was consistent with the observed values. Results of the SSGWAS using the genomic relationship matrix and all SNP with MAF ≥0.10 were consistent with the BB GWAS. None of the SNP reached the Bonferroni-corrected 5% significance level for WFEC or PFEC EBV. The Q-Q and Manhattan plots for the SSGWAS are shown in Supplementary Figures S1, S2.

The RHM detected one 500-SNP window located between 65.9 and 69.9 Mb on chromosome 5 that reached Bonferroni-corrected genome-wide significance for PFEC EBV. Five additional adjacent, overlapping windows on chromosome 5 achieved suggestive significance for PFEC EBV, and one of the windows achieved suggestive significance for WFEC EBV (Table 2). All six of these regions were within the region of interest identified by the BB analysis (Figure 3). The RHM also identified a 500-SNP region on chromosome 16 that reached a suggestive level of significance for WFEC EBV and corresponded to regions identified by the BB analysis (Table 2). Three overlapping regions on chromosome 20 also achieved suggestive significance for WFEC EBV (Table 2). This region was not previously identified in the BB analysis and did not correspond to the candidate region on chromosome 20 in Table 1.

**TABLE 2:** Summary statistics of the regional heritability analysis using 500-SNP windows.

EBV	Chromosome	Position, Mb		Heritability		LRT	−logP	Significance
		Start	End	Regional	Total			
PFEC	5	59.3	62.5	0.20	0.87	9.47	3.24	Suggestive
	5	60.9	64.3	0.21	0.86	10.68	3.53	Suggestive
	5	62.5	65.9	0.22	0.89	9.77	3.32	Suggestive
	5	64.3	68.0	0.31	0.87	12.14	3.88	Suggestive
	5	65.6	69.9	0.40	0.79	14.72	4.48	5%
	5	68.0	71.7	0.31	0.73	10.75	3.55	Suggestive
WFEC	5	60.8	64.3	0.16	0.89	8.06	2.90	Suggestive
	16	44.7	49.5	0.22	0.99	7.78	2.83	Suggestive
	20	40.3	43.6	0.40	0.98	8.78	3.08	Suggestive
	20	42.0	45.0	0.38	1.00	12.85	4.04	Suggestive
	20	43.6	46.9	0.40	1.00	9.38	3.22	Suggestive

EBV, estimated breeding value; LRT, likelihood ratio test; −log *P* = negative logarithm of nominal (i.e., uncorrected) *p*-value; PFEC, postweaning fecal egg count; WFEC, weaning fecal egg count. Values of −log *p* greater than 3.51 and 2.81 correspond to Bonferroni-corrected genome-wide significance levels of *p* < 0.05 and *p* < 0.10, respectively.

## 4 Discussion

This study provided evidence of an association between PFEC EBV in progeny-tested Katahdin rams and a series of SNP on ovine chromosome 5. The study involved only 84 rams, but FEC EBV were derived from 3,272 phenotyped progeny from 14 flocks.

For PFEC, nominal significance levels of 7.56 × 10^–7^–3.46 × 10^–4^ and Bonferroni-corrected chromosome-wide BB significance levels of *p* < 0.05 were achieved for the most significant SNP in 12 adjacent 1-Mb bins on chromosome 5. However, after Bonferroni adjustment, none of the SNP achieved genome-wide significance in either the bin-based analysis in ASREML or the individual-SNP analysis in GCTA. Results of single-marker regression analysis do not, however, explicitly consider clumping of significant, or near-significant, SNP in specific regions of the genome. Localization of influential SNP in specific regions can result from linkage of closely associated SNP with one or more QTL. However, the presence of apparently influential SNP across a 12-Mb region on chromosome 5 could not be explained by simple LD with a single influential QTL. For an additive model of QTL effects, patterns of LD across this region (Figure 4) instead indicated the presence of three potential QTL within the region.

Regional heritability mapping was used to assess associations between specific regions of the genome and PFEC EBV. Regional heritability mapping is inferior to single-marker GWAS for detection and localization of individual QTL but facilitates identification of potentially important regions of the genome. Regional heritability mapping has been shown to be superior to single-marker GWAS for detection of genomic regions that contain multiple QTL with small to modest individual additive or epistatic effects (Uemoto et al., 2013; Shirali et al., 2016). Results of RHM indicated that the region of interest on chromosome 5 accounted for a substantial proportion of the observed variation in PFEC EBV. An association with parasite resistance in sheep has not been previously reported for this region of chromosome 5. However, a study of Katahdin lambs using a 50K Illumina SNP array (Becker et al., 2020) identified a SNP at 73.0 Mb on ovine chromosome 5 that achieved an association with FEC with a nominal significance level of 5.23 × 10^–6^ from a single-locus linear regression model. However, this SNP had only a suggestive genome-wide significance level (*p* < 0.10) in those data.

The power of RHM to detect putative QTL loci was demonstrated in an analysis of EBV for milk production using 2,590 Japanese Holstein bulls (Gervais et al., 2017). In that study, single-SNP regression identified significant SNP for milk and fat yield only on chromosome 14, in the region known to be associated with the *DGAT1* gene (Grisart et al., 2002). No significant SNP were identified for protein yield. By contrast, RHM identified a significant region common to all three traits on bovine chromosome 14, as well as significant regions for fat yield on chromosome 5 and protein yield on chromosome 18.

In sheep, Al Kalaldeh et al. (2019) analyzed FEC in a multi-breed population of 7,339 Australian lambs. Single-SNP regression identified only three significant SNP, all within a 3.4 Mb region on chromosome 2 and with false-discovery rates (FDR) of 0.02–0.03. Regional heritability mapping with 200, 500 or 1,000 SNP windows all confirmed a significant association with FEC for corresponding regions on chromosome 2. The 1,000-SNP RHM allowed coalescence of the previously identified SNP into a single 12.2 Mb region with a FDR of ≤0.003. The RHM also identified three progressively overlapping 1,000-SNP windows in a 9.7 Mb region of chromosome 6 with FDR of ≤0.019.

Comprehensive meta-analyses of GWAS of parasite resistance in sheep have not yet been conducted. However, Riggio et al. (2014) used RHM for meta-analysis of three studies involving Scottish Blackface (Riggio et al., 2013), Sarda x Lacaune (Sechi et al., 2009), and Martinik Blackbelly x Romane (Sallé et al., 2012) lambs. The studies differed in the type of infection (natural versus artificial) and the predominant helminth genera (predominantly Strongyle nematodes versus a combination of Strongyle and *Nematodirus* species). The RHM supported a role for a region within the ovine MHC on chromosome 20 across the three studies. A significant region was also identified on chromosome 14 but appeared to be specific to Nematodirus FEC.

In the current study, large minor-allele frequencies in the region of interest on chromosome 5 suggested that favorable SNP alleles were either segregating at relatively high frequencies in more than one parent breed or were derived primarily from one of the ancestral breeds. Minor allele effects were consistently negative (i.e., favorable for FEC) within the region of interest, and, based on these results, we hypothesized, but could not test further, that favored chromosome segments on chromosome 5 may have originated in the parasite-resistant hair sheep ancestor of the Katahdin breed. We acknowledge, however, that the power to detect informative markers is positively associated with MAF and that our design simply may not have been able to detect informative markers that were present at lower MAF.

The region between 59.6 and 71.0 Mb on chromosome 5 that was identified in our study contains several candidate genes that may affect innate and acquired immunity. Among well-characterized functional genes, *IL12B* at 68.5 Mb on chromosome 5 codes for the p40 subunit of both interleukin (IL)-12 and IL-23. Structural changes in *IL12B* thus can affect the function of both IL-12 and IL-23. These cytokines promote T-helper (Th) type 1 cell differentiation and secretion of IFNγ (Watford et al., 2004). Changes in *IL12B* that result in down-regulation of IFNγ may therefore favor Th2 cell differentiation. Administration of anti-IL-23 antibodies prevented implantation of intestinal nematodes in mice (Gomez-Samblas et al., 2018).

Hair sheep lambs of primarily St. Croix ancestry that were artificially infected with *H. contortus* at 120–150 days of age and compared to lambs of a composite (50% Dorset, 25% Rambouillet, 25% Finnish Landrace) wool-sheep line had lower expression of *IL12A* (i.e., the p35 subunit of IL-12 and IL-23) in abomasal tissue and lower expression of *IFNγ* in lymph nodes at 3 days after infection (MacKinnon et al., 2009). These results, and those of the current study, suggest that stimulation of a Th2 immune response and down-regulation of IFNγ may be achieved by the action of genetic variants that influence secretion of IL-12 and IL-23.

A second functional gene, *IL17B*, is located at 58.2 Mb on chromosome 5, just outside the core 12-bin region on chromosome 5 associated with PFEC EBV but within the larger 19-bin region that contained 16 SNP markers that achieved BB chromosome-wide significance. *IL17B* codes for IL-17B, which is a member of the IL-17 cytokine superfamily (Bie et al., 2017) and is involved in the Th17 pattern of T-cell differentiation. The role of IL-17 and associated Th17 cells in sheep is gradually emerging (McRae et al., 2015). In mice, a Th17 immune response was required for the hypercontractility of intestinal muscle involved in expulsion of *Trichinella spiralis* from the gastrointestinal tract (Steel et al., 2019).

The region in question also contains regulatory genes that may influence immune function. The early B-cell transcription factor (*Ebf-1*) gene at 73.2 Mb 5 codes for early B-cell transcription factor 1 (EBF1). In the mouse, EBF1 is essential for B-cell commitment, development and maintenance, and immune responses were severely reduced by *Ebf1* inactivation (Vilagos et al., 2012). SNP variants in *Ebf1* were likewise associated with IL-12 p40 blood levels in humans with Stage I melanoma, and SNP in both *IL17B* and *Ebf1* were associated with melanoma susceptibility and patient outcomes (Fang et al., 2015). *Ebf1* regulates genes by activation and repression, modulating chromatin structure, and histone dimethylation (Treiber et al., 2010; Hagman et al., 2012; Boller and Grosschedl, 2014).

*Larp1*, at 63.7 Mb on ovine chromosome 5, interacted with *Mtorc1* to influence T-cell metabolism and differentiation and control early B-cell development, survival, and metabolism in mice (Iwata et al., 2016). Differences in expression of the *CCR4-NOT* transcription complex subunit 8 (*Cnot8*) gene, located at 63.8 Mb on ovine chromosome 5, were observed in association with intestinal inflammation associated with inflammatory bowel disease in humans (Yuan et al., 2017).

The hydroxytryptamine receptor 4 (*Htr4*) gene is located at 57.6 Mb on ovine chromosome 5. Hydroxytryptamine (5-HT), commonly known as serotonin, is stored at peripheral sites in mast cells and released following cross-linking with immunoglobulin E. Stimulation of HTR4 enhanced release of IL-1β and IL-8 and reduced secretion of IL-12 and TNF-α in mature human dendritic cells (Idzko et al., 2004).

The inducible T-cell kinase (*Itk*) gene is located at 66.1 Mb on ovine chromosome 5 and is essential for various T-cell functions, especially during a Th2 response in mice (Broussard et al., 2006) and humans (Ghosh et al., 2018). A key role has also been shown for *Itk* in production of both IFNγ and IL-4 by natural killer cells in mice (Au-Yeung and Fowell, 2007). Expression of the cytoplasmic FMR1 interacting protein 2 (*Cyfip2*) gene, which is adjacent to *Itk* at 66.4 Mb on chromosome 5, was associated with T-cell adhesion in humans (Mayne et al., 2004).

Another interesting candidate gene is glutamate ionotropic receptor AMPA type subunit 1 (*Gria1*). *Gria1* is located at 62.5 Mb on ovine chromosome 5 and associated with asparaginase hypersensitivity in humans (Chen et al., 2010). The context of that study was to investigate the genetic basis for allergic reactions which occur in up to 45% of patients receiving asparaginase derived from *Escherichia coli* or *Erwinia chrysanthemi* for treatment of childhood acute lymphoblastic leukemia. Asparaginase is present in the cuticle of several helminth parasites and was proposed as a vehicle for inducing immunity to *Dirofilaria immitis*, which causes heartworm disease in dogs (Chandrashekar and Tsuji, 2000). These studies provide a potential mechanism whereby genetic variation in asparaginase sensitivity could influence parasite immunity.

In contrast to results for chromosome 5, no obvious candidate genes were present in the region of chromosome 16 associated with WFEC EBV. The region between 46.7 and 51.7 Mb contains genes that code for three members of the cadhedrin superfamily (*Cdh9*, *Cdh10*, and *Cdh12*) which, in common with other protocadhedrins, mediate cell-cell adhesion. Protocadhedrins have not been shown to have an explicit role in parasite immunity but are involved in cellular apoptosis (Pancho et al., 2020).

In the current study, power to detect significant marker associations with FEC by GWAS was limited by the small number of available sires but was to some extent compensated for by use of EBV (rather than individual phenotypes) to reduce residual errors associated with individual observations and by focusing on SNP with relatively high MAF. The latter approach excluded rare genomic variants from consideration but was justified in this relatively recently formed composite breed in order to focus on the impacts of higher-frequency alleles contributed by parasite-resistant and parasite-susceptible founder breeds. In addition, RHM provided opportunity to account for apparent clumping of marginally significant individual SNP on chromosome 5, thereby detecting a significant influence on FEC for a candidate region that had not been previously identified but included potentially important functional and regulatory genes. We acknowledge that high Type II error may have accounted, in part, for our failure to detect significant effects for any of our five *a priori* candidate regions. We also acknowledge that limited power to detect a full spectrum of informative SNP likely contributed to the apparent dominant role of chromosome 5 in accounting for additive variation in PFEC. However, our results nevertheless unambiguously supported an association with GIN resistance in Katahdin sheep for the identified region on chromosome 5. Further study of this region is necessary to identify specific candidate loci that affect parasite resistance. Results in Figure 3 suggest the presence of three separate informative SNP in the region, but more complex causal relationships involving epistatic interactions among structural and regulatory loci may also be present.

Apparent inconsistencies among studies designed to identify informative SNP markers for resistance to GIN in sheep can arise from insufficient power to detect widely distributed QTL variants with small to modest individual effects on GIN resistance or from differences among breeds in the genetic architecture controlling GIN resistance. Our attempt to accommodate our limited number of available progeny-tested sires by *a priori* identification of candidate regions associated with GIN resistance from the literature was not successful. This outcome was consistent with previous assertions (McManus et al., 2014; Al Kalaldeh et al., 2019) that GIN resistance is mainly under polygenic control. A review of the genetics of helminth resistance in sheep (Karrow et al., 2014) highlights the myriad functional and regulatory pathways that are potentially involved in GIN resistance and provides conceptual support for both polygenic control and potential breed differences in genetic control of GIN resistance. Among livestock species, methods for genomic prediction are most highly developed for milk production in Holstein dairy cattle (Jiang et al., 2019), with predictions of additive genetic merit based on relatively dense SNP arrays to properly identify and weight putative associations with largely anonymous QTL. Yet even here, relatively few influential SNP markers have been unambiguously associated with specific functional or regulatory loci (Weller et al., 2018), and multi-breed genomic evaluations are still being developed (Van Raden et al., 2020; Cesarani et al., 2022). These results suggest that genomic prediction of additive genetic merit for GIN resistance in sheep will require SNP arrays that provide relatively comprehensive coverage of the genome and have sufficient power to address potential breed-specific structuring of informative markers and underlying QTL.

## Data Availability

The datasets presented in this study can be found in online repositories. The names of the repository/repositories and accession number(s) can be found below: Animal-GRIN; https://agrin.ars.usda.gov/main_webpage_dev/ars.
