# Supplementary material for: Single Nucleotide Polymorphism Effects on Lamb Fecal Egg Count Estimated Breeding Values in Progeny-Tested Katahdin Sires
Source: Front Genet. 2022 May 3;13:866176. doi: 10.3389/fgene.2022.866176 (PMC9110833; doi:10.3389/fgene.2022.866176)
Supplement: Supplementary file 1 [file DataSheet2.docx]

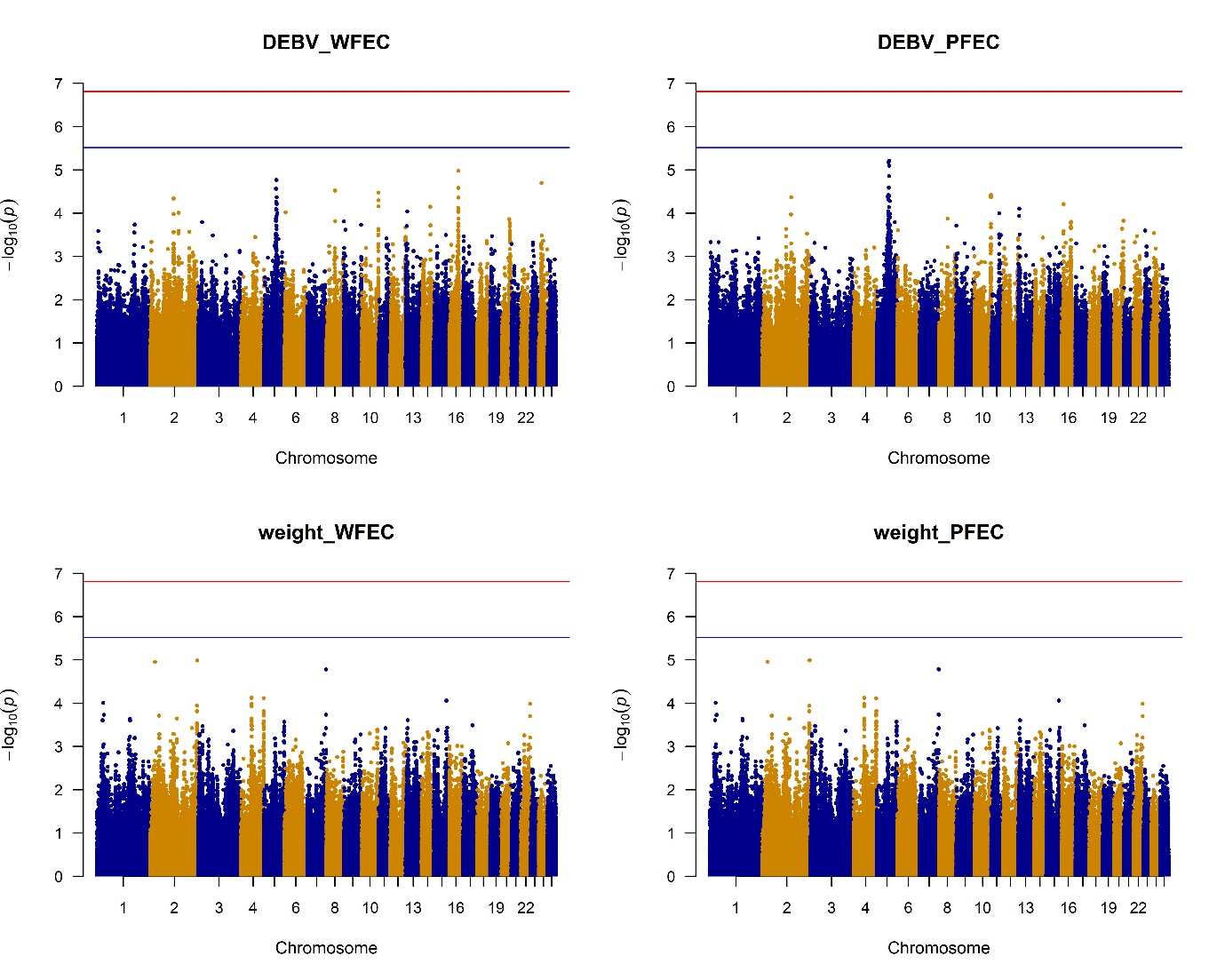


**Supplemental Fig. 2** Manhattan plots from the single-SNP genome-wide association study for deregressed weaning and postweaning fecal egg count estimated breeding values (DEBV_WFEC and DEBV_PFEC, respectively) using a genomic animal relationship matrix. The red line represents Bonferroni-corrected significant (P ≤ 0.05) level (-log *P* = 6.81) and blue line represent the suggestive (P ≤ 0.10) significance level (-log *P* = 5.51).
