# Supplementary material for: Single Nucleotide Polymorphism Effects on Lamb Fecal Egg Count Estimated Breeding Values in Progeny-Tested Katahdin Sires
Source: Front Genet. 2022 May 3;13:866176. doi: 10.3389/fgene.2022.866176 (PMC9110833; doi:10.3389/fgene.2022.866176)
Supplement: Supplementary file 2 [file DataSheet1.docx]

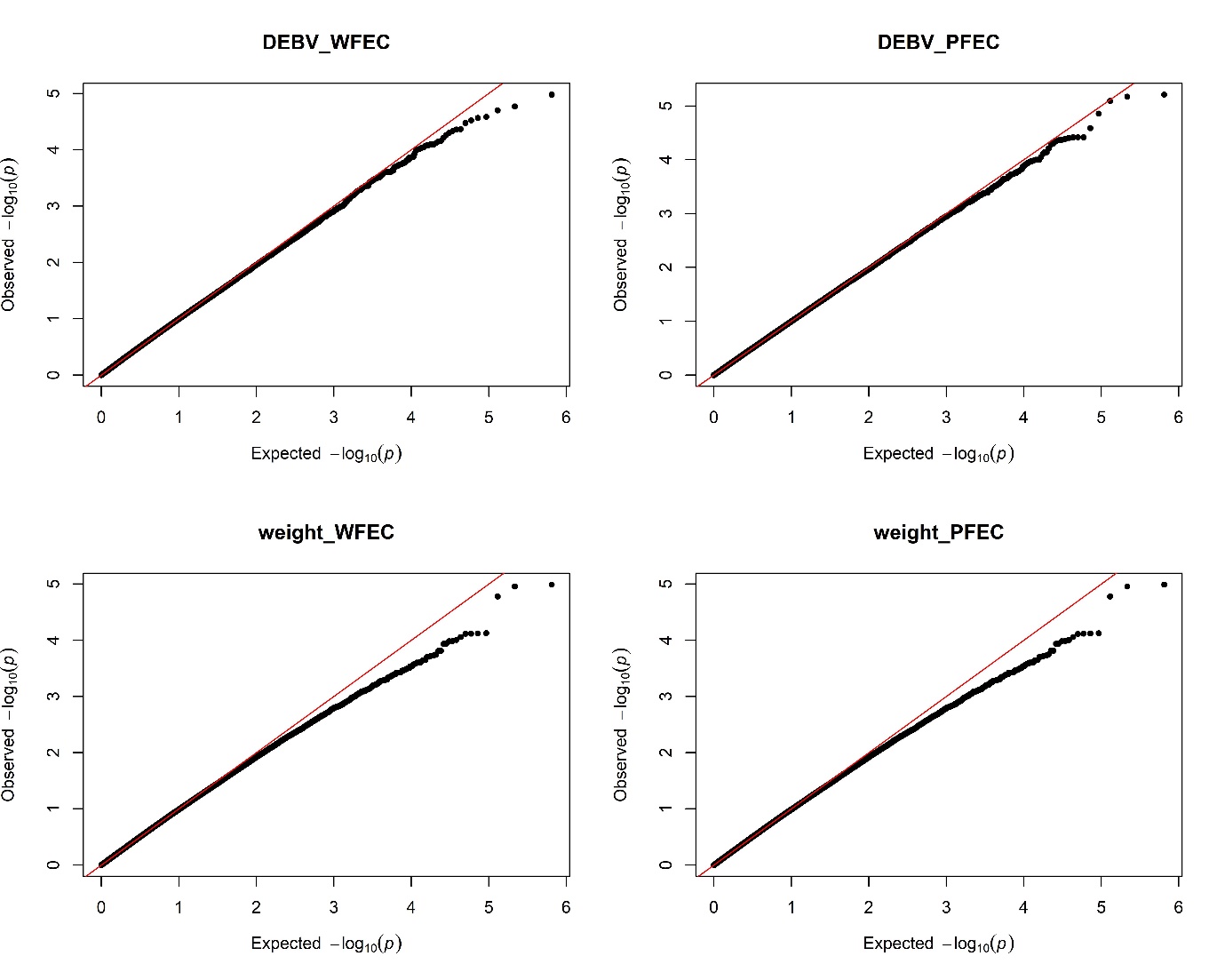


**Supplemental Fig. 1** Q-Q plots showing the relationship between the observed and expected values for -Log_10_(P) for deregressed weaning and postweaning fecal egg count estimated breeding values (DEBV_WFEC and DEBV_PFEC, respectively) from the single-SNP genome-wide association study using a genomic animal relationship matrix. P is the nominal significance level for each SNP.
